# A Comparison of Radiometric and Spectrometric Emissivity Evaluation Methods in Infrared Thermometry

**DOI:** 10.3390/s26051671

**Published:** 2026-03-06

**Authors:** Vid Mlačnik, Igor Pušnik

**Affiliations:** Faculty of Electrical Engineering, University of Ljubljana, 1000 Ljubljana, Slovenia; vid.mlacnik@fe.uni-lj.si

**Keywords:** radiation thermometry, emissivity, spectral emissivity, effective emissivity, emissivity evaluation, real-body thermometry

## Abstract

**Highlights:**

**What is the main finding?**

- Instrumental emissivity is a prerequisite in infrared thermometry and can be traceably obtained either by a spectrometric or radiometric method.

**What are the implications of the main findings?**

- In practical comparison, results correspond between the two methods for samples of sufficient quality.
- A radiometric method is practically preferable for simplicity, inexpensiveness and generally lower uncertainty of subsequent temperature measurement.

**Abstract:**

Accurate radiation thermometry of real objects critically depends on knowledge of surface emissivity, which is rarely known a priori and often varies with surface condition, temperature, and environment. Although theoretical models for spectral emissivity evaluation exist, their practical validation under application-relevant conditions remains limited. In this study, spectral and radiometric emissivity evaluation methods are compared on metallic samples up to 350 °C. The spectral method derives effective emissivity from spectroscopy-measured spectral emissivity using instrument-specific spectral sensitivity (responsivity), while the radiometric method evaluates emissivity directly from radiance measurements using a radiation thermometer and a reference contact temperature. The radiometric method is treated as an application-level reference. Stable and homogeneous chromium nitride (CrN)-coated samples show good agreement between the two methods, whereas raw metals and polysiloxane-coated samples highlight practical limitations related to sample surface instability and inhomogeneity. The results demonstrate that spectral emissivity evaluation is valid in practice when its underlying method assumptions are fulfilled, while radiometric evaluation remains preferable for in situ infrared thermometry.

## 1. Introduction

In radiation thermometry, surface emissivity is a dominant source of uncertainty in non-contact temperature measurements of real objects. Unlike ideal blackbodies, real surfaces exhibit emissivity values that are spectrally dependent, spatially non-uniform, sensitive to oxidation, roughness, coatings, and temperature. As a result, emissivity cannot be treated as a fixed material constant but must be evaluated for a specific surface under given measurement conditions.

Current industrial and field applications of radiation thermometry almost exclusively involve real bodies rather than blackbodies. For broadband radiation thermometers and thermal imagers, the detected signal represents a spectrally weighted integral of emitted and reflected radiation, making accurate emissivity compensation essential for traceable temperature measurements. For this, gray-body radiation thermometry is widely implemented in commercial instruments; however, the traceability of emissivity compensation is not inherently guaranteed for real bodies [1].

In practical infrared thermometry, emissivity values are frequently adopted from the literature rather than evaluated as part of the measurement procedure. Commercial and handbook emissivity tables provide representative radiometrically obtained emissivity values for a wide range of materials and surface conditions and are widely used in industrial and field applications [2,3,4,5]. Similarly, numerous experimental studies rely on emissivity values taken from the literature when direct emissivity evaluation is impractical or outside the scope of the investigation [6,7,8,9,10]; however, it is often unclear how these values were obtained, which instrument spectral sensitivity (within the spectral range) they correspond to, and what the uncertainty they were evaluated at is. In situ emissivity evaluations are therefore preferred for practical emissivity evaluations in the field of traceable radiation thermometry.

Two general emissivity evaluation methods are commonly used: spectral emissivity evaluation methods based on infrared spectroscopy, and radiometric emissivity evaluation methods based on direct radiance measurements with radiation thermometers. Although both approaches are experimental in nature, they differ fundamentally in their measured quantities. The radiometric method is considered an application-level reference, as radiometric quantities are obtained exactly as they would be in radiation thermometry. In the spectral emissivity evaluation method, theoretical foundations for spectral emissivity evaluation and effective emissivity formulation have been established in the literature [1,11]. However, experimental validation of these models under application-relevant conditions remains limited. The aim of this study is therefore to experimentally compare spectral and radiometric emissivity evaluation methods on identical samples and to assess their consistency, limitations, and impact on subsequent temperature measurement accuracy.

### 1.1. Current Practices in Spectral Emissivity Evaluation Methods

In spectral emissivity evaluation methods, wavelength-resolved emissivity is measured using infrared spectroscopy, typically with Fourier transform infrared (FTIR) spectrometers. The measured spectral emissivity is subsequently processed to obtain an effective emissivity corresponding to the spectral sensitivity (responsivity) of a given radiation thermometer.

Simplified approaches often rely on averaging spectral emissivity over the nominal spectral band of the instrument, while neglecting detailed spectral variability. Chrzanowski [11] introduced the concept of effective emissivity derived from spectral data by explicitly accounting for spectral weighting. A comprehensive spectral radiation model for radiation thermometry, including instrument-specific effective emissivity, was further developed in [1]. These works provide the theoretical foundation for spectral emissivity evaluation. However, the practical accuracy of this approach depends on the calibration and uncertainty of the FTIR spectrometer, particularly for hemispherical reflectance-based emissivity measurements, where the uncertainty budget is often not fully specified.

### 1.2. Current Practices in Radiometric Emissivity Evaluation Methods

Radiometric emissivity evaluation methods determine emissivity directly from radiance measurements performed with a radiation thermometer. The emissivity is evaluated by comparing the measured surface radiance with the blackbody radiance at the same temperature, obtained with a radiation thermometer or calculated from contact temperature measurement.

Various application-oriented radiometric approaches have been reported in the literature [12,13,14,15]. Riou [16] derives equations and demonstrates a radiometric effective emissivity evaluation method using a radiation thermometer and heater with an integrated observation slot for radiance measurements. As the sample surface in this heater is replaceable, this method is practically preferable due to its simplicity and low equipment cost. Because emissivity is derived from the same radiometric signal used for temperature determination, radiometric emissivity evaluation yields emissivity values directly compatible with practical infrared thermometry. For this reason, the radiometric emissivity evaluation method is treated in this study as an application-level reference.

## 2. Methods for Evaluation of Effective Emissivity

### 2.1. Theoretical Definition of Spectral Evaluation of the Effective Emissivity

Previous theoretical developments [3,4] have shown that spectral emissivity $\varepsilon \left(\lambda \right)$ (defined over the radiant spectrum in wavelength λ) can be converted into an effective emissivity $\varepsilon_{eff}\left(T\right)$, specific to a radiation thermometer, by spectral integration over the instrument spectral sensitivity $S\left(\lambda \right)$. This formulation enables an additional method of effective emissivity evaluation; however, a direct comparison between spectrally and radiometrically evaluated emissivity is required to evaluate correspondence. The effective emissivity $\varepsilon_{eff}\left(T\right)$ is obtained by weighting the spectral emissivity with the spectral sensitivity of the radiation thermometer $S\left(\lambda \right)$ and integrating over the wavelength range (1) [1,11].

(1) $$\varepsilon_{eff}\left(T\right)=\frac{\int {\varepsilon \left(\lambda \right)M}_{BB}(T,\lambda )S\left(\lambda \right)d\lambda }{\int M_{BB}\left(T,\lambda \right)S\left(\lambda \right)d\lambda }$$

In the spectral approach, thermal emission $M_{BB}(T,\lambda )$ is calculated using Planck’s law (2).

(2) $$M_{BB}(T,\lambda )=\frac{2hc^{2}}{\lambda^{5}}{\left[e^{\frac{hc}{\lambda k_{B}T}}-1\right]}^{-1}$$

Here, h is the Planck constant, $k_{B}$ is the Boltzmann constant, c is the speed of light in vacuum, λ is the wavelength, and T is the absolute temperature. Spectral integration in this study is performed in 0.01 μm discrete wavelength increments.

### 2.2. Theoretical Definition of Radiometric Evaluation of Effective Emissivity

The radiometrically evaluated effective emissivity is obtained from the direct radiation model expressed in Equation (3). The total radiance detected by a radiation thermometer $M_{meas}\left(T\right)$ is composed of the thermal emission of the observed surface $\varepsilon \left(\lambda \right)M_{BB}\left(T,\lambda \right)$ and the reflected radiance $\left(1-\varepsilon \left(\lambda \right)\right)\;M_{refl}\left(\lambda \right)$ originating from the environment, as previously derived in [1], and weighted by the spectral sensitivity of the radiation thermometer $S\left(\lambda \right)$.

(3) $$M_{meas}\left(T\right)=\int (\varepsilon (\lambda )\;M_{BB}(T,\lambda )\;S(\lambda )+(1-\varepsilon (\lambda ))\;M_{refl}(\lambda )\;S(\lambda ))d\lambda$$

For readability, the blackbody radiance detected by the radiation thermometer is expressed using the blackbody sensor (BBS) radiance characteristic $M_{BBS}\left(T\right)$, as in Equation (4).

(4) $$M_{BBS}\left(T\right)=\int M_{BB}(T,\lambda )S\left(\lambda \right)\;d\lambda$$

Replacing the spectral emissivity ε(λ) with the equivalent effective emissivity $\varepsilon_{eff}$ allows the radiometric model to be expressed in a compact form, from which the effective emissivity can be directly evaluated, as shown in Equation (5).

(5) $$\varepsilon_{eff}\left(T\right)=\frac{M_{meas}-M_{refl}}{M_{BBS}\left(T\right)-M_{refl}}$$

In practical measurement conditions, the terms in Equation (5) correspond to the radiance measured by a radiation thermometer when observing the real surface $M_{meas}$ and the environment $M_{refl}$, and to the radiance of a blackbody with the same temperature as the measured surface. The blackbody radiance is obtained using the previously characterized blackbody sensor radiance characteristic (BBS) at the reference temperature, which corresponds to the true surface temperature of the emissivity sample, independently measured by a contact thermometer behind the sample surface.

### 2.3. Blackbody Sensor (BBS) Characteristic Function

The blackbody sensor (BBS) characteristic represents the instrument-specific mapping between blackbody temperature and detected radiance of a radiation thermometer. It inherently accounts for the spectral integration of blackbody radiance weighted by the spectral sensitivity of the detector and therefore provides a direct relationship between temperature and measured radiance for a given instrument. In practice, the BBS characteristic is obtained experimentally from the radiation thermometer if radiance output is supported, or by calculating the detected blackbody radiance of a specific radiation thermometer using numerical integration of Planck’s law over the instrument’s spectral sensitivity. Common analytical approximations, such as the Sakuma–Hattori method, rely on an effective wavelength and bandwidth; however, these generalized approximations disregard the detailed distribution of the detector spectral sensitivity and are therefore not fully adapted to a specific instrument.

**Figure 1:**
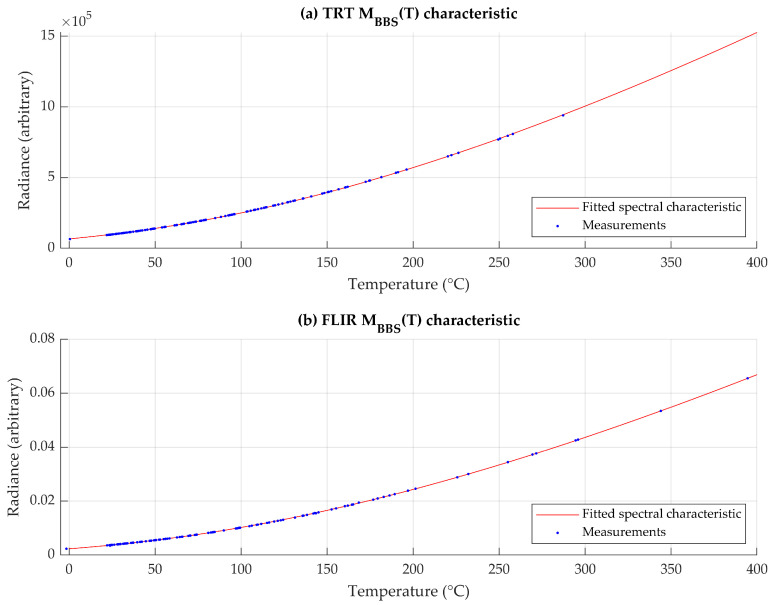
Blackbody radiance characteristic, as detected by the sensor, for (**a**) Heitronics TRT II and (**b**) FLIR T1020.

To obtain a more reliable characteristic, the BBS function was determined by direct numerical integration according to Equation (4), and subsequently scaled to instrumental units using experimental calibration data. As both radiance and blackbody temperature measurements were available, the scale of the simulated characteristic was adjusted using a least-squares fit, resulting in an instrument-specific BBS characteristic (Figure 1). The BBS characteristic was initially evaluated in discrete temperature steps, while piecewise linear interpolation was applied for subsequent conversions.

In addition to the forward characteristic $M_{BBS}\left(T\right)$, the inverse characteristic $T_{BBS}\left(M\right)$ was derived, enabling conversion between radiance-based and temperature-based quantities. This bidirectional mapping is required for the consistent propagation of uncertainty contributions expressed in temperature units, correction application to measured radiance and contact temperature to equivalent blackbody radiance measurement conversion.

**Figure 2:**
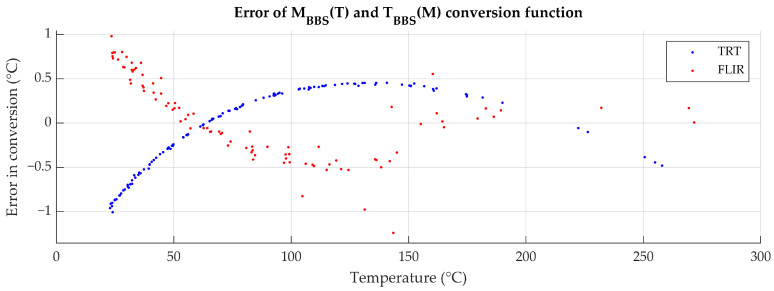
Error of derived $M_{BBS}\left(T\right)$ and, consequently, $T_{BBS}\left(M\right)$ characteristic in comparison to embedded instrumental mapping.

Errors of conversion in Figure 2 were calculated by comparing experimentally measured radiances to those calculated from matching temperature measurements by the use of $M_{BBS}\left(T\right)$. While the absolute error of conversion is significant, the numeric error of its inverse, when nesting one function into another (conversion of T to M and back to T) for the purpose of uncertainty calculations, is negligible, in the range of 10^−14^ °C.

Calibration corrections provided in the calibration certificates of the radiation thermometers were applied to measurements in temperature units. For this, the measured radiance was first converted to an equivalent temperature using the inverse BBS characteristic. The correction was then applied in the temperature domain, and subsequently corrected temperatures were converted back to corrected radiances using the forward BBS characteristic.

Conversion errors of nested BBS characteristic conversion were evaluated to be in the range of 10^−14^ °C and are considered negligible in this study.

### 2.4. Spectral Emissivity Measurements Using Fourier Transform Infrared (FTIR) Spectrometer

**Figure 3:**
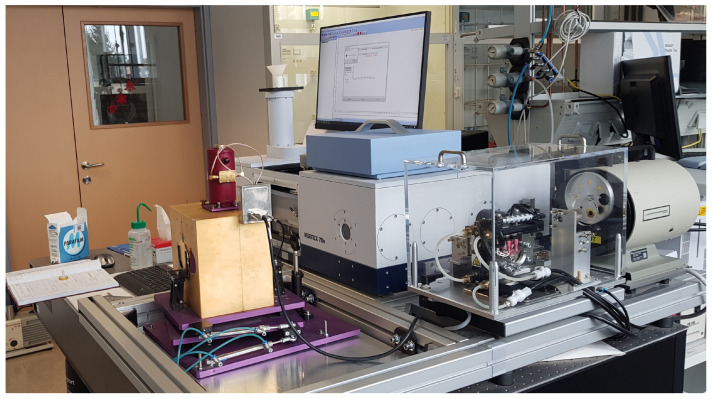
The Bruker Vortex 70v with gold hemispherical reflectivity measurement modification.

Spectral emissivity was derived from FTIR reflectance measurements under the assumption of surface opacity, using the relation ε(λ)=1−ρ(λ). The measurements were performed using a Vertex v70 FTIR spectrometer (Bruker Optics GmbH, Ettlingen, Germany) equipped with a thermal infrared configuration, a MCT detector, and a gold-coated integrating sphere (Figure 3).

A full uncertainty budget for the reflectance-derived emissivity could not be found for the specific device; however, it is annually calibrated by the manufacturer. Typical uncertainty associated with emissivity derived from FTIR reflectance measurements using similar spectrometers has been investigated in inter-laboratory comparison (ILC) studies involving FTIR systems comparable to the setup used in this work. In one such ILC, in which an equivalent (though not identical) Bruker Vertex FTIR spectrometer with a similar detector and integrating-sphere configuration participated, deviations of up to approximately 4.6% in emissivity were reported in the 8–14 µm spectral range [17]. This value may therefore be regarded as a representative estimate of the typical uncertainty associated with reflectance-based spectral emissivity measurements using such FTIR configurations. Another inter-laboratory comparison study employing optimized FTIR measurement setups has demonstrated smaller emissivity uncertainties, in the order of ±1% [18,19]. These results illustrate the level of uncertainty that can be achieved under carefully optimized conditions, but are not necessarily representative of routine measurement configurations.

### 2.5. Radiometric Evaluation Procedure for Effective Emissivity

**Figure 4:**
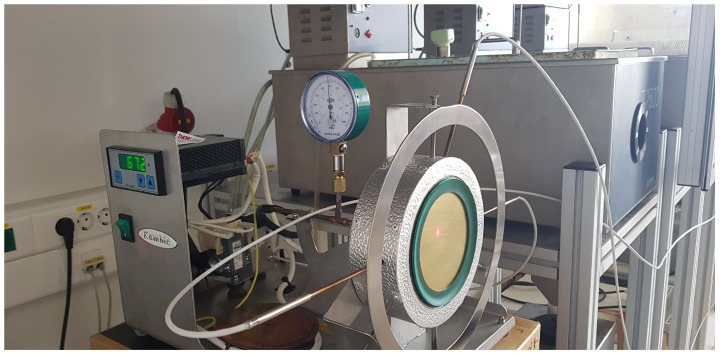
Flat-plate calibrator with the vacuum-mounted sample of brass and the rubber gasket.

The radiometric evaluation procedure for effective emissivity was conducted in a controlled laboratory enviroment using a flat-plate calibration prototype (Kambič d.o.o., Semič, Slovenia) with exchangeable sample plates, shown in Figure 4. The samples were mounted on a heated copper block using a vacuum pump to ensure good thermal contact, uniform heating and stable thermal conditions.

**Figure 5:**
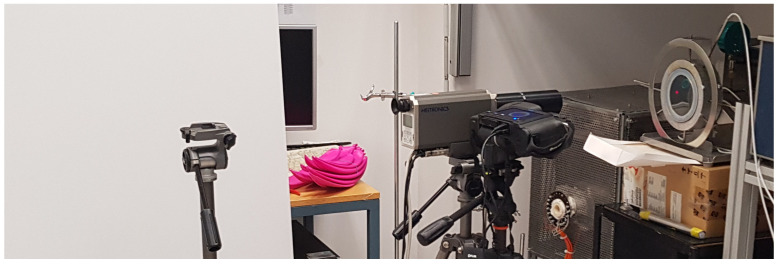
Measurement setup for radiometric emissivity evaluation.

Radiance from the sample surface was measured using a radiation thermometer positioned nearly normal to the surface (Figure 5). Environmental radiance was controlled by a plastic sheet of high emissivity, positioned behind radiation thermometers in the direction of specular reflection. Environmental temperature and its stability were monitored with a radiation thermometer. While the influence of atmosphere, namely the humidity, was found to be negligible for these radiation thermometers in [20], the air temperature and the relative humidity were monitored with the calibrated climate monitor to verify sufficient stability.

The reference surface temperature was obtained using calibrated contact thermometers placed in thermal wells beneath the emitting surface. The heat flux to the surface q was determined from temperature measurements at two different depths z within the copper heater block. Using the thermal conductivity of copper $\lambda_{Cu}$, the heat flux was calculated according to Fourier’s law, as shown in Equation (6).

(6) $$q=-\lambda_{Cu}\;\frac{T(z_{2})-T(z_{1})}{z_{2}-z_{1}}$$

Using the determined heat flux, the surface temperature was corrected according to Equation (7) by accounting for heat conduction through the copper block, the substrate, and, where applicable, the coating layer.

(7) $$\Delta T=q\;\sum \frac{\Delta z_{i}}{\lambda_{i}}$$

**Figure 6:**
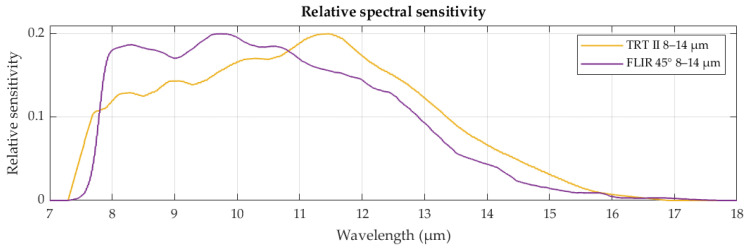
Relative spectral sensitivities of Heitronics TRT II and FLIR T1020.

Radiation thermometers, used in this study, are Heitronics TRT II radiation thermometer (HEITRONICS Infrarot Messtechnik GmbH, Wiesbaden, Germany) and FLIR T1020 thermal camera (FLIR Systems AB, Täby, Sweden). The operating spectral range of both devices is nominally the same, 8–14 μm, but their sensors operate under different physical principles and use different absorption materials. As evident from Figure 6, their relative spectral sensitivities are not equally spectrally distributed. The spectral sensitivity data for Heitronics TRT II and FLIR T1020 were obtained from manufacturers in graph form and digitized.

**Figure 7:**
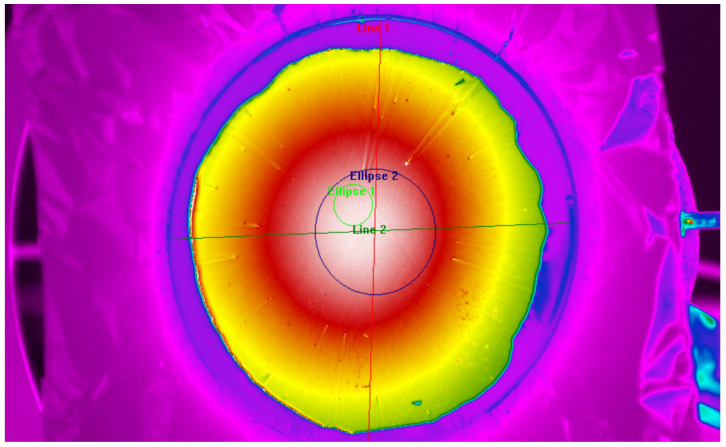
Regions of interest in radiometric effective emissivity measurements. The green circle (Ellipse 1) corresponds to radiation thermometer region of interest and the blue circle (Ellipse 2) corresponds to the wider area of homogeneity measurement.

Regions of interest (ROI) were defined on thermal images using ResearchIR 4.40.11 thermal image analysis software (FLIR Systems AB, Täby, Sweden) to evaluate sample spatial radiance inhomogeneity and to extract representative mean radiance values for subsequent analysis. Measurements were performed in a region of interest, 1 cm from the center of the sample (Figure 7, green circle) in order to decrease the influence of varying thickness of the coating, as well as to avoid potential thermal inhomogeneity above the vacuum line at the center of the flat-plate calibrator.

Another set of measurements was conducted over a wider central circle with a diameter of 40 mm (Figure 7, blue circle), to capture the homogeneity of radiance over the region of interest and its immediate vicinity. The radiant homogeneity of the wider area was included in the uncertainty calculation to account for possible alignment errors between repeated spectral and radiometric evaluations of emissivity.

Effective emissivity was evaluated from measured radiance values by accounting for the sample radiance, the environmental radiance, and the blackbody sensor (BBS) radiance corresponding to the temperature of the gradient-corrected reference thermometer measurement according to Equations (4)–(7).

### 2.6. Calculation of Uncertainty of the Radiometrically Evaluated Emissivity

The uncertainty of the radiometrically evaluated emissivity was determined by propagation of individual radiant uncertainty contributions derived from the input uncertainties using a deviation-based analysis. The effective emissivity was evaluated for deviated input parameters, and the difference between the nominal and deviated results was taken as the corresponding output uncertainty contribution.

For clarity, input uncertainties were expressed in temperature units, while the uncertainty propagation itself was performed in the radiance domain.

The blackbody radiance $M_{BBS}\left(T\right)$ was calculated from the contact temperature T, with the uncertainty of conversion $U_{BBS}$ in units of temperature, calculated from errors, displayed in Figure 2. The uncertainty contribution is calculated using Equation (8).

(8) $$U_{\varepsilon (U_{BBS})}\left(T\right)=\frac{M_{sample}\left(T\right)-M_{env}}{M_{BBS}\left(T\pm U_{BBS}\right)-M_{env}}-\varepsilon_{eff}\left(T\right)$$

The uncertainty of the sample radiance measurement $U_{M_{sample}}$ is the uncertainty of the RT measurement. The uncertainty contribution is calculated using Equation (9).

(9) $$U_{\varepsilon (U_{M_{sample}})}\left(T\right)=\frac{\left(M_{sample}\pm U_{M_{sample}}\right)-M_{env}}{M_{BBS}\left(T\right)-M_{env}}-\varepsilon_{eff}\left(T\right)$$

The uncertainty of the sample radiance measurement consists of the calibration uncertainty of radiation thermometers, combined with the homogeneity of radiance in the region of interest (ROI), measured from an individual thermal image to account for the radiant inhomogeneity of a sample. The input uncertainty contributions of sample radiance and radiance measurement are combined according to Equation (10), as they both impact the same quantities.

(10) $$U_{M_{sample}}=\sqrt{{U_{RT}}^{2}+{U_{ROI\;homog}}^{2}}$$

The uncertainty of environmental radiance contains the evaluated radiant temperature inhomogeneity of the hemispherical environment of the surface, as well as the calibration uncertainty of radiation thermometers. The uncertainty contribution is calculated using Equation (11).

(11) $$U_{\varepsilon \left(U_{T_{env}}\right)}\left(T\right)=\frac{M_{sample}-M_{BBS}\left(T_{BBS}\left(M_{env}\right)\pm U_{T_{env}}\right)}{M_{BBS}\left(T\right)-M_{BBS}\left(T_{BBS}\left(M_{env}\right)\pm U_{T_{env}}\right)}-\varepsilon_{eff}\left(T\right)$$

**Figure 8:**
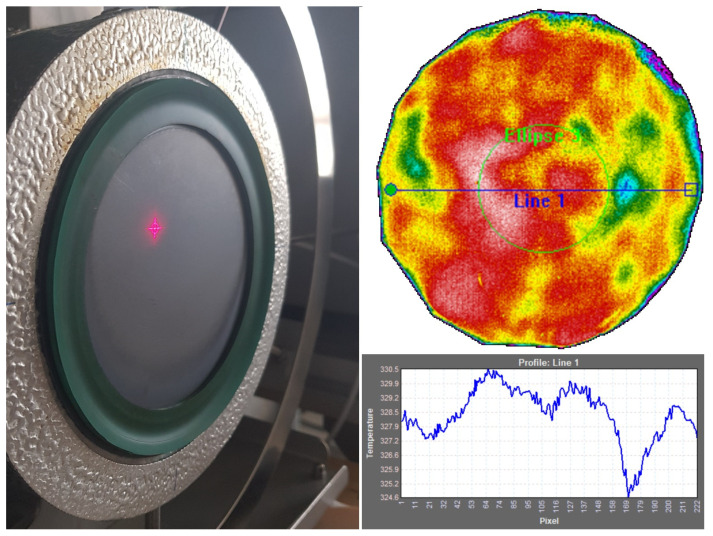
Homogeneity evaluation using black sample (**left**) and thermal homogeneity profile along Line 1 in the thermal image (**right**).

The uncertainty of true temperature $U_{T}$ accounts for the uncertainty of the contact thermometer (PRT) and any temperature difference between the contact thermometer and the sample surface in the region of interest, namely, the evaluated temperature homogeneity of the ROI on the sample during measurements. Accuracy, stability and homogeneity of calibrator surface temperature were evaluated using a sample of reference emissivity paint. Temperature homogeneity was quantified by evaluating the spatial variation in temperature within the ROI (Figure 8), while surface temperature and temporal stability were characterized from repeated measurements over the acquisition period, also accounting for the stability of thermal gradient compensation. The expanded uncertainty of surface temperature stability and homogeneity was evaluated to be 1.5 °C at temperatures up to 350 °C.

The true temperature directly affects $M_{BBS}\left(T\right)$, as well as $M_{sample}$, by the change in radiant emission, according to Planck’s law. The uncertainty contribution is calculated using Equation (12).

(12) $$U_{\varepsilon (U_{T})}\left(T\right)=\frac{M_{BBS}\left(T_{BBS}\left(M_{sample}\right)\pm U_{T}\right)-M_{env}}{M_{BBS}\left(T\pm U_{T}\right)-M_{env}}-\varepsilon_{eff}\left(T\right)$$

The expanded input uncertainties in radiometric emissivity evaluation are listed in Table 1. Typical expanded radiant homogeneity uncertainties ranged between 0.5% and 1.5% of the surface temperature.

The combined measurement uncertainty of the effective emissivity was calculated as the geometric sum of the individual uncertainty contributions following Equation (13).

(13) $$U_{\varepsilon }\left(T\right)=\sqrt{{U_{\varepsilon (U_{BBS})}}^{2}\left(T\right)+{U_{\varepsilon (U_{M_{sample}})}}^{2}\left(T\right)+{U_{\varepsilon \left(U_{T_{env}}\right)}}^{2}\left(T\right)+{U_{\varepsilon (U_{T})}}^{2}\left(T\right)}$$

## 3. Results

### 3.1. Effective Emissivity of CrN-Coated Samples

**Figure 9:**
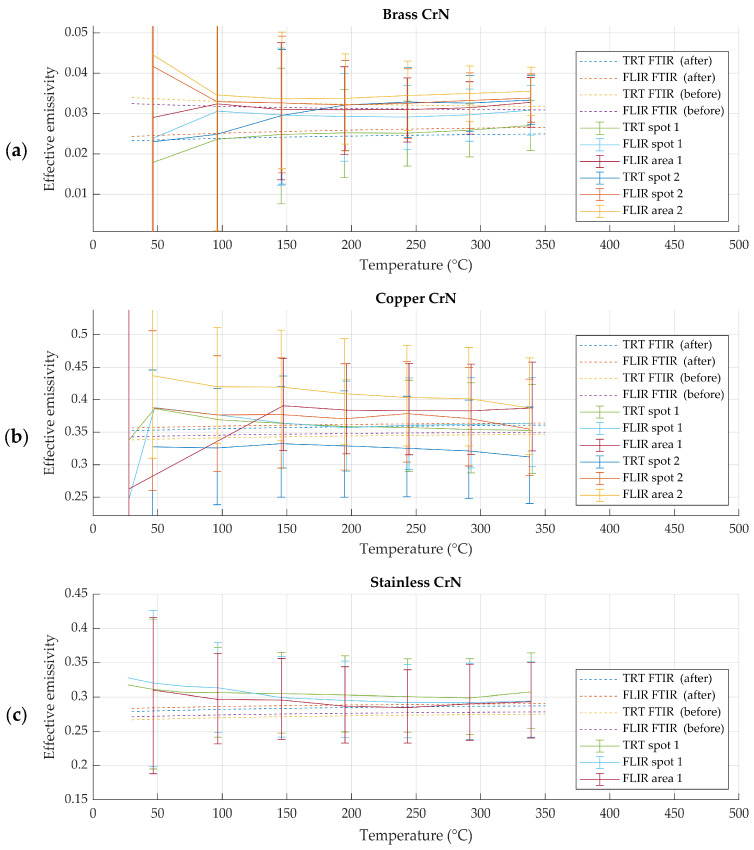
Spectral and radiometric effective emissivity results for CrN-coated samples of (**a**) brass, (**b**) copper and (**c**) stainless steel. Spot refers to narrower region of interest and area refers to wider region of interest, while uncertainties in both account for inhomogeneity of wider region of interest.

Chromium nitride (CrN)-coated metallic samples exhibited high thermal stability and spatial homogeneity during repeated heating cycles up to 350 °C. No visible degradation or temporal drift of spectral emissivity was observed for these samples over the duration of the experiments. Effective emissivity values obtained using the spectral emissivity evaluation method and the radiometric emissivity evaluation method showed good agreement within the expanded uncertainty of the radiometric method across the investigated temperature range. This agreement was observed consistently for both radiation thermometers used in the study, as evident in Figure 9.

**Figure 10:**
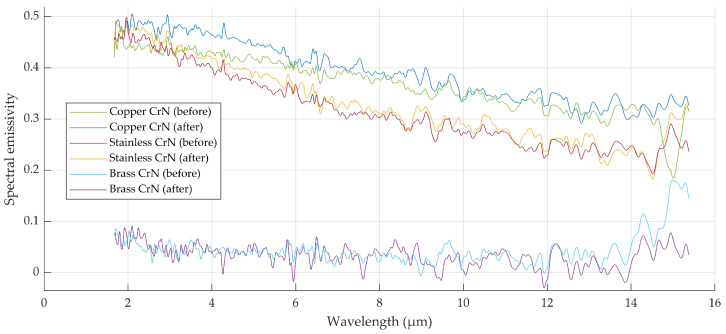
Spectral emissivities of copper, stainless steel and brass samples with chromium-nitride (CrN) coating.

For CrN-coated samples of brass substrate, a systematic deviation in emissivity values was observed. While the emissivity remained stable with temperature and between repeated measurements, its absolute value was consistently lower than that obtained for CrN coatings on copper and stainless steel substrates. This behavior was observed using both radiometric and spectral emissivity evaluation methods, as evident in Figure 9 and Figure 10.

The authors attribute inconsistent effective emissivity to differences in surface finish of the polished substrates, which were observed in profilometric measurements.

### 3.2. Effective Emissivity of Raw Metallic Samples

**Figure 11:**
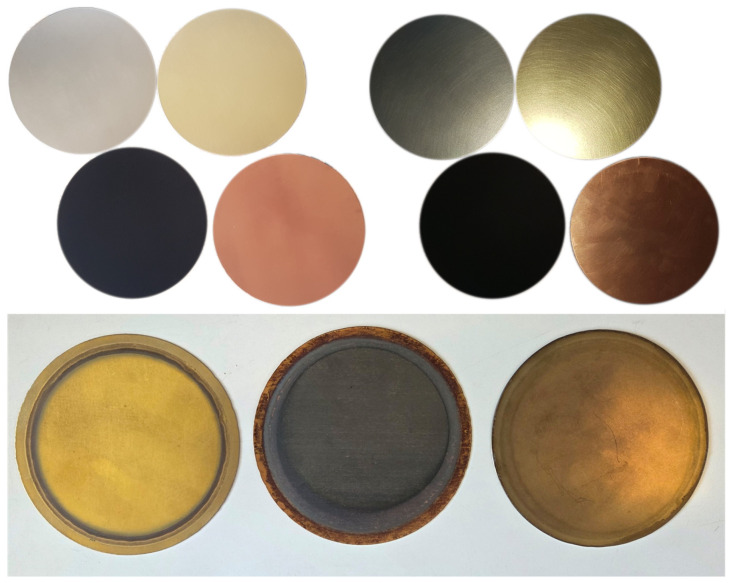
Samples of raw materials (brass, copper, stainless steel) before (**above**) and after exposure to increased temperature (**bellow**).

Raw metallic samples exhibited visible changes in surface condition during thermal exposure (Figure 11). In particular, copper samples showed a pronounced increase in effective emissivity with increasing temperature and repeated heating cycles. Stainless steel and brass samples exhibited smaller but measurable variations in effective emissivity.

**Figure 12:**
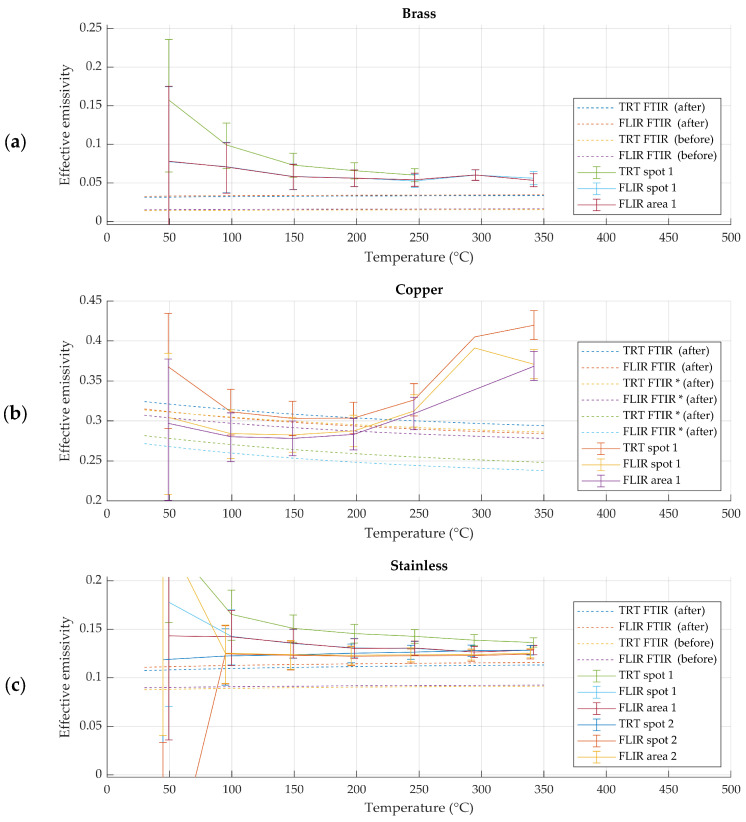
Spectral and radiometric effective emissivity results for fresh and polished (**a**) brass, (**b**) copper and (**c**) stainless steel. Spot refers to narrower region of interest and area refers to wider region of interest, while uncertainties in both account for inhomogeneity of wider region of interest. Measurements marked with * are repeated measurements.

For raw metallic samples, effective emissivity values obtained using spectral and radiometric methods did not consistently agree within the expanded uncertainty of the radiometric method. The discrepancies between materials and measurement repetitions are shown in Figure 12.

### 3.3. Effective Emissivity of Polysiloxane-Coated Samples

Polysiloxane-coated samples exhibited pronounced spatial variations in effective emissivity. These variations were consistent with the observed thickness gradients of the applied coating.

**Figure 13:**
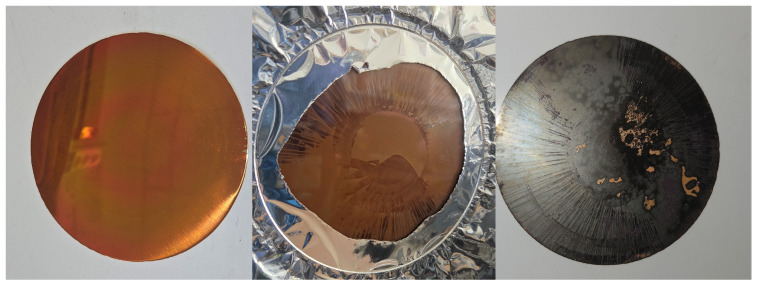
The copper sample with polysiloxane–spinel coating before, after the first, and after the third round of heating.

Effective emissivity values obtained using spectral and radiometric evaluation methods on these samples showed significant discrepancies. In most cases, differences exceeded the expanded uncertainty of the radiometric method. Repeated measurements indicated limited reproducibility, particularly due to inconsistent coating thickness and substrate instability, visible in Figure 13, where coating interference produces a colorful effect on a fresh sample surface, whereas the diffuse oxidation is clearly visible after thermal exposure.

The effective emissivity results exhibited similar behavior as those of raw metals, with additional mismatch between repeated measurements and methods due to placement and inhomogeneity inconsistencies.

### 3.4. Impact on Temperature Measurement

**Figure 14:**
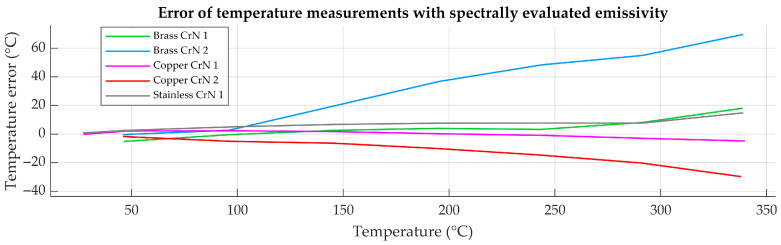
Error of temperature measurement with spectrally evaluated effective emissivity when compared to radiometric method as an application-level reference.

Differences in effective emissivity obtained using the spectral and radiometric evaluation methods resulted in corresponding differences in calculated surface temperature. The magnitude of temperature measurement uncertainty in Figure 14 depended strongly on the absolute emissivity value and its uncertainty. For low-emissivity samples, small differences in emissivity produced large deviations in calculated temperature, whereas for higher-emissivity samples, the impact was reduced.

**Figure 15:**
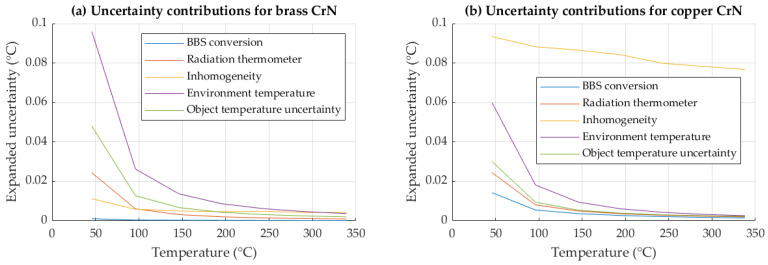
Uncertainty contributions in effective emissivity evaluation for (**a**) brass and (**b**) copper substrate, both coated with CrN.

An analysis of uncertainty contributions of the reference (radiometric) method is shown in Figure 15.

For the radiometric emissivity evaluation method, the effective emissivity, determined at elevated temperatures, exhibited lower theoretical uncertainty due to constant input contributions. As follows from Equation (5), the contribution of reflected radiation to the measured signal becomes increasingly negligible at higher temperatures, since the radiance emitted by the surface dominates the emissivity expression. Consequently, the relative influence of reflected environmental radiation on the evaluated emissivity—and its associated uncertainty—decreases with increasing temperature. Similarly, all other contributions with constant (temperature-independent) input uncertainties also become less significant. As emissivity is a ratio of radiance signals, a characterization at elevated temperatures yields a higher signal-to-noise ratio. The resulting uncertainty contributions are consequently relatively lower at higher temperatures, improving the method uncertainty.

## 4. Discussion

The results demonstrate that agreement between spectral and radiometric emissivity evaluation methods critically depends on surface stability, homogeneity, and the validity of the underlying modeling assumptions. For CrN-coated metallic samples, which exhibited high thermal stability and spatial homogeneity, effective emissivity values obtained by both methods were consistent within the expanded uncertainty of the radiometric approach. This agreement suggests the validity of the spectral emissivity evaluation framework established in [1,11] under application-relevant conditions.

In contrast, raw metallic samples and polysiloxane-coated samples exhibited behavior that violates key assumptions of both emissivity evaluation methods. Oxidation, surface evolution, and pronounced spatial inhomogeneity resulted in emissivity values that were not stable or representative of the emitting surface as a whole. Under such conditions, discrepancies between spectral and radiometric emissivity evaluation do not indicate a failure of either method but rather reflect limitations imposed by the physical properties of the samples.

A systematic difference was observed for CrN-coated brass samples, which exhibited lower effective emissivity compared to CrN coatings on copper and stainless steel substrates. This observation indicates that the emissivity of coated surfaces may not be entirely independent of the substrate. Differences in substrate composition, surface chemistry, and microstructure can influence coating growth and morphology. For sufficiently thin coatings, partial optical interaction with the substrate cannot be excluded, resulting in an effective emissivity that reflects combined coating–substrate behavior rather than an intrinsic property of the coating material alone.

The accuracy of radiometric emissivity evaluation further depends on the determination of the true surface temperature. In the present study, the surface temperature was obtained by linear extrapolation of the measured temperature gradient within the heater–sample assembly, using temperature measurements from multiple thermal wells located at different depths beneath the emitting surface. The extrapolation accounted for the known thermal conductivities of the copper heater block, the metallic substrate, and, where present, the coating layer.

The impact of emissivity uncertainty on temperature measurement is strongly non-linear, particularly for low-emissivity surfaces. As demonstrated by the uncertainty analysis, small deviations in emissivity can result in large temperature errors when emissivity approaches zero.

Regarding broader applicability, both spectral and radiometric emissivity evaluation methods are most reliable for thermally stable, homogeneous, and optically opaque surfaces. For materials with porous structure, low thermal conductivity, or pronounced surface roughness, such as many ceramics, polymers, or composite materials, additional challenges arise, including appropriate mounting methods, subsurface heat transport, spatial emissivity variations, and increased temperature gradients. While emissivity evaluation remains formally possible for such materials, its practical accuracy is reduced unless additional characterization or correction steps are implemented.

## 5. Conclusions

This study investigated the applicability of a spectrally resolved emissivity evaluation method based on FTIR measurements by comparison with a radiometric reference method. The results demonstrate that, for stable and homogeneous surfaces, the effective emissivity derived from spectral data is consistent with radiometrically evaluated emissivity within the combined measurement uncertainty.

Chromium nitride (CrN) coatings exhibited the highest level of agreement between the two methods across the investigated temperature range, confirming their suitability as validation samples for emissivity evaluation. For these samples, the correlation between spectral and radiometric results remained well within the uncertainty limits, supporting the validity of the spectral-to-effective emissivity formulation.

In contrast, samples exhibiting surface instability or inhomogeneity, such as polysiloxane-coated and uncoated metallic substrates, showed increased variability and reduced agreement between methods as well as repeated measurements. These results highlight the practical limitations of emissivity evaluation for materials affected by oxidation, coating degradation, or spatial non-uniformity, rather than deficiencies of the evaluation methods themselves.

The findings confirm that spectrally resolved emissivity data, when properly integrated using instrument-specific spectral sensitivity, can provide reliable effective emissivity values for radiation thermometry. However, careful consideration of material stability, surface homogeneity, and environmental influences remains essential for accurate emissivity evaluation in practical applications.

## Figures and Tables

**Table 1:** Expanded input uncertainties of the radiometric effective emissivity evaluation result.

Uncertainty Contribution	Symbol	Expanded Uncertainty (°C)
Blackbody radiance calculation	$U_{BBS}$	1.0 °C
Radiance measurement	$U_{M_{sample}}$	$\sqrt{{0.6\;{}^{\circ}C}^{2}+{U_{ROI\;homog}}^{2}}$
Environmental radiance measurement	$U_{T_{env}}$	3.0 °C
Uncertainty of true temperature	$U_{T}$	1.5 °C

## Data Availability

The original contributions presented in this study are included in the article. Further inquiries can be directed to the corresponding author.

## Abbreviations

The following abbreviations are used in this manuscript:

ILC	Inter-laboratory comparison
BB	Blackbody (or corresponding characteristic function)
BBS	Blackbody sensor characteristic function
FTIR	Fourier transform infrared (spectroscopy)
PRT	Platinum resistance thermometer
RT	Radiation thermometer
ROI	Region of interest
CrN	Chromium nitride
